# Dr. R. C. Fisher’s Fasting Cure

**Published:** 1887-05

**Authors:** Hannah McL. Shepard


### DR. R. C. FISHER'S FASTING CURE.

Of the many innovations, new departures and reforms in medical science or curative practice, not any, since the introduction of Homeopathy, is so marked as that known as the "Fasting Cure," discovered and now practiced with almost miraculously effective results by Dr.

Richard C. Fisher, of Washington, D. C. His method of treatment comes near to fulfilling the prophesy that in the enlightened future, medicine will be dispensed with, and nature be allowed to make use of her own processes entirely to rid herself of the ills to which, only when her benign laws are transgressed, "flesh is heir."

It was during the time when Dr. Fisher was walking the Larzaretto, in San Francisco, that he began to seek after some means of getting the diseased human system into condition for the effective action of curative agents. After lengthened study and close observation of hospital cases his conclusion was that the desired end could be attained only by getting a complete control of the digestive organs, and under that control eliminating from the system the obstructions lying in the way of cure. Further study and experiment convinced him that this result could be attained in no other way than by abstainance from food until the body was thoroughly cleansed of the gross and effete matters which clog liver and intestines and form the body of adipose deposit. To secure this result, however, such a lengthened fast would be required that the patient would be in danger of the fate of the unlucky horse whose master attempted to train to live on an oat *per diem.* Therefore, some agent must be found by which to sustain vitality during the eliminating process of the fast. This agent, the Doctor after a time happily discovered, and his patients by taking three teaspoonfuls *per diem* of an almost tasteless, and, as he says, exceedingly simple liquid are sustained in such strength during fasts, of from ten to forty days, that they are enabled to attend to their daily avocations and to walk from three to ten miles daily.

Dr. Fisher's success in mastering chronic diseases of long standing, has been almost unparalleled, and he has made the happy discovery that the fast itself with the accompanying steam baths and open-air exercise, upon which he insists, is usually sufficient, nature herself taking advantage of improved conditions and perfecting the cure.

If, as some think, to cure disease is to "cast out devils," and the souls most truly in prison are those confined in disordered bodies, then in counteracting abnormal conditions and undoing the fetters of disease, a most truly holy work is accomplished. Several persons with whom the writer has conversed affirm, and her own experience confirms the statement, that not only is the body freed from detrimental conditions and restored to health, but tone and vigor are given to the mind, and the spiritual nature is clarified and uplifted.

After observing the fast and the subsequent dietary rules imposed by the Doctor's system, it is found that the appetites have returned to their normal condition. The senses of smell and taste become acute. The fine flavor of fruits and simple foods is fully enjoyed, highly-seasoned and rich viands and spirituous liquors are distasteful and repulsive. Numerous cases could be cited where patients have been cured of the tobacco habit and of alcoholism, and one amusing and singular fact is on record of a lady who from her childhood had heen addicted to biting her nails—even to the quick—who, during the period of a twenty-three day's fast, discovered that she had relinquished the bad practice, neither has she, since her recovery, resumed it.

Among the many cures effected since the Doctor came to Washington, in November last, are three cases of cancer of the stomach and one of cancer of the tongue.

The following recent cases of treatment are given, as fair examples of the practice. In a pronounced case of diabetes the patient has not only recovered from that distressing and dangerous malady, there being now absolutely no sugar in the urine, and all other symptoms of the disease having disappeared, but the sight of one of her eyes, which was entirely lost, has returned. Two eminent oculists, who examined the diseased organ, affirmed that the vitrous humor was solidified and that no cure was possible. Persons desiring further authentication of this statement can secure it by addressing Fred. E. Brown, Esq., No. 143 Worth Street, New York City.

Miss Anne Held, a patient now under treatment for catarrh of more than ten years' standing, recovered the sense of smell on the fifth day of her fast, it having been totally lost for seven years. Miss Held fasted fifteen days, and is now living upon fruit and bread for a few weeks while the cure is being perfected.

Mr. D. A. Cronan, No. 37 Riggs Market, Washington, D. C., was afflicted for more than two years with a falling sickness, which seized him every day and many times a day. He was cured by a ten day's fast. The Doctor has on his list of cures three similar cases.

In rheumatism, especially that of the chronic kind, the cures are simply marvellous. In reference to one ease, Dr. Krouse, a physician of excellent standing in this city, said that Dr. Fisher had done more to cure it in two days than he had been able to do in six months.

I may be excused for saying a few words in regard to my own case. I had been suffering from rheumatism for years, and at the time of commencing treatment with Dr. Fisher was greatly debilitated with malaria. I had a bad cough and physicians of high standing here and in New York found my lungs badly diseased with tuberculosis. I fasted ten days. I attended to my business—a government desk—every day, and walked from two to five miles a day. This was in January. I am now—April 7th—in better health than I have been in ten years. I have no rheumatism ; my cough is almost gone, and I believe that the trouble in my lungs is steadily decreasing.

HANNAH McL. SHEPARD.

